# ER-associated CTRP1 regulates mitochondrial fission via interaction with DRP1

**DOI:** 10.1038/s12276-021-00701-z

**Published:** 2021-11-26

**Authors:** Seong Keun Sonn, Seungwoon Seo, Jaemoon Yang, Ki Sook Oh, Hsiuchen Chen, David C. Chan, Kunsoo Rhee, Kyung S. Lee, Young Yang, Goo Taeg Oh

**Affiliations:** 1 Department of Life Science, Heart-Immune-Brain Network Research Center, Ewha Woman’s University, Seoul, 03760 Republic of Korea; 2 Department of Radiology, Yonsei University, Seoul, 120-752 Republic of Korea; 3 Department of Life Science, Research Center for Women’s Disease, Sookmyung Women’s University, Seoul, Republic of Korea; 4 Division of Biology, California Institute of Technology, Pasadena, CA 91125 USA; 5 Department of Biological Sciences, Seoul National University, Seoul, Republic of Korea; 6 Laboratory of Metabolism, Center for Cancer Research, National Cancer Institute (NCI), Bethesda, MD 20892 USA

**Keywords:** Endoplasmic reticulum, Mitochondria

## Abstract

C1q/TNF-related protein 1 (CTRP1) is a CTRP family member that has collagenous and globular C1q-like domains. The secreted form of CTRP1 is known to be associated with cardiovascular and metabolic diseases, but its cellular roles have not yet been elucidated. Here, we showed that cytosolic CTRP1 localizes to the endoplasmic reticulum (ER) membrane and that knockout or depletion of CTRP1 leads to mitochondrial fission defects, as demonstrated by mitochondrial elongation. Mitochondrial fission events are known to occur through an interaction between mitochondria and the ER, but we do not know whether the ER and/or its associated proteins participate directly in the entire mitochondrial fission event. Interestingly, we herein showed that ablation of CTRP1 suppresses the recruitment of DRP1 to mitochondria and provided evidence suggesting that the ER–mitochondrion interaction is required for the proper regulation of mitochondrial morphology. We further report that CTRP1 inactivation-induced mitochondrial fission defects induce apoptotic resistance and neuronal degeneration, which are also associated with ablation of DRP1. These results demonstrate for the first time that cytosolic CTRP1 is an ER transmembrane protein that acts as a key regulator of mitochondrial fission, providing new insight into the etiology of metabolic and neurodegenerative disorders.

## Metabolism: When mitochondria grow too big

A protein named CTRP1 helps mitochondria, the powerhouses of cells, to divide after they grow too big, and could provide insight into neurodegenerative disorders such as Alzheimer’s disease. CTRP1 is known to be involved in metabolic and cardiovascular diseases. The finding that it is also highly expressed in mitochondria-rich tissues such as heart muscle led Goo Taeg Oh at Ewha Womans University and Young Yang at Sookmyung Women’s University, both in South Korea, and coworkers to further investigate its function. They found that CTRP1 is highly expressed in the cell’s transport system, where it helps to regulate mitochondrial division. CTRP1 deficiency in mice produced abnormally long mitochondria, causing cells to become resistant to programmed cell death, and leading to degeneration of neurons. These results may increase understanding of diseases related to mitochondrial defects.

## Introduction

CTRP1 belongs to the C1q/TNF family, the members of which have four structurally distinct domains: a signal peptide at the N-terminus, a short variable region, a collagenous domain and a C-terminal globular domain. CTRP1 levels were reportedly increased in the plasma or sera of patients with type II diabetes and ob/ob mice^1–3^. Circulating CTRP1 levels were also found to be increased in the sera of patients with coronary artery disease^4–8^ and heart disease^9^, suggesting that this protein has proatherogenic and cardiac effects. CTRP1 has been shown to regulate vascular function^10–12^ and protect against acute ischemic injury in the heart^13^. CTRP1 levels were reportedly increased in the sera of hypertensive patients, and CTRP1 has been shown to stimulate aldosterone production and plays a role in blood pressure homeostasis^14,15^. These findings suggest that the secreted form of CTRP1 plays important roles in regulating metabolic and cardiovascular functions. Regarding other family members, CTRP3 appears to promote energy production by inducing mitochondrial ROS, upregulating the expression of PGC-1α in vascular smooth muscle cells and inducing mitochondrial biogenesis in cardiomyocytes^16,17^. CTRP5 was increased in mitochondrial DNA-depleted myocytes and was shown to activate AMP-activated protein kinase^18^. Interestingly, Innamorati and colleagues suggested that GIF (G protein-interacting protein, which is another name for the above-described CTRP1) has a putative transmembrane domain and is highly expressed in the mitochondria-rich heart and other muscles^19^. This result suggested that cytosolic CTRP1 may modulate mitochondrial functions to regulate metabolic and cardiovascular functions. However, the cellular and molecular functions of cytosolic CTRP1 in mitochondrial dynamics have not yet been determined.

The dysregulation of mitochondrial fusion/fission dynamics has been associated with physiological effects, such as aging, as well as pathologies such as cardiovascular disease, metabolic disease and neurodegenerative diseases (e.g., Parkinson’s, Huntington’s and Alzheimer’s diseases)^20–22^. In the fission process, DRP1 oligomerizes and drives mitochondrial fission at mitochondrial fission sites^23,24^. MFF, MiD49 (also known as MIEF1) and MiD51 (also known as MIEF2) regulate mitochondrial localization and the GTPase-mediated activation of DRP1^25,26^. Although numerous DRP1 puncta localize to mitochondrial constriction sites, mitochondrial fission occurs infrequently, suggesting that additional events may be required to complete the fission process. The ER and mitochondria form extensive contacts with one another and show close dynamic interactions. ER tubules wrap around mitochondrial constrictions and fission sites^27^. Two proteins, ER-bound inverted formin (FH2 and WH2 domain-containing; IFN2) and mitochondria-bound spire 1C, contribute to mitochondrial constriction through actin polymerization and thereby support DRP1 oligomerization^28,29^. However, no previous study has identified an ER protein that is directly associated with DRP1 assembly and affects mitochondrial fission. The literature indicates that DRP1 deficiency induces apoptotic resistance by influencing Bax oligomerization at the mitochondrial fission site^30^. Moreover, the DRP1 adaptors MiD49 and MiD51 have been shown to be essential for crista remodeling during apoptosis, and ablation of MFF, MiD49 and MiD51 was shown to affect resistance to cell death^25,26^. A central process in apoptosis is the release of cytochrome c from mitochondrial cristae into the cytoplasm. This release, which occurs through the mitochondrial outer membrane pores generated by BAX and BAK, initiates caspase cascade activation, leading to apoptosis^31,32^.

In this study, we examined the detailed role of cytosolic CTRP1. Our novel results show that CTRP1 localizes to the ER membrane and regulates mitochondrial fission through an interaction with the mitochondrial fission protein DRP1. Based on this finding, we further assessed whether CTRP1 plays a critical role in mitochondrial fission by examining the functional interaction between CTRP1 and DRP1 at ER–mitochondria contact sites (EMCSs).

## Materials and methods

### Mice

*Ctrp1*^fl/fl^ mice were generated through a standard gene-targeting strategy using embryonic stem cells (clone E14). The targeting vector for the conditional allele was from the Knockout Mouse Project (KOMP) and contained a neomycin-resistance marker flanked by FRT and *loxP* sites that were inserted next to exons 2 and 4 of *Ctrp1*. Mice were backcrossed more than seven times onto the C57BL/6J background (Jackson Laboratory) and were crossed to a Flp recombinase–expressing transgenic mouse strain (Jackson Laboratory). FRT recombination was confirmed by PCR. *Pcp2*–*Cre*;*Ctrp1*^fl/fl^ and *Alb*–*Cre*;*Ctrp1*^fl/fl^ mice were generated by crossing *Ctrp1*^fl/fl^ mice with *Pcp2*–*Cre* or *Alb*–*Cre* transgenic strains of mice (Jackson Laboratory). The success of the crosses was confirmed by PCR. The PCR primers used were as follows: *loxP* site primer: LR—5′-ACT GAT GGC GAG CTC AGA CC-3′; genomic primer: DF—5′-GCA CAC CTG TAT ACC AGA CA-3′, genomic primer: DF2—5′**-**GCA CAC CTG TAT ACC AGA CA-3′; genomic primer: DR—5′**-**GAG GGA GAG AAG AAA GCC TA-3′. All animal care and experimental procedures were performed in compliance with protocols approved by the Institutional Animal Care and Use Committee of Ewha Woman’s University.

### Plasmids and RNAi oligonucleotides

GFP–CTRP1, BFP–CTRP1, CTRP1–Flag, Flag-tagged CTRP1 deletion mutant (1–98, 1–138, 99–281, 139–281, 20–281 and 31–281), and Myc- and His-tagged CTRP1 deletion mutant (1–98, 1–138, 99–281, 139–281, 20–281 and 31–281) proteins were generated by PCR amplification of full-length *CTRP1* (isoform 1; NCBI accession number NM_030968.3) and cloning of the product into the EcoRI–BamHI sites or BamHI–EcoRI sites of the pEGFP-N3 (Clontech), pEGFP-C1 (Clontech), pCMV-Tag 4A (Stratagene) or pcDNA.3.1/Myc-His(–) A (Invitrogen) plasmids. Point mutations in *CTRP1* (for expression of CTRP1^C13R^ and CTRP1^C13A^) were generated with a Phusion Site-directed Mutagenesis Kit according to the manufacturer’s protocol (Thermo Fisher Scientific, Inc.). The construct encoding prohibitin–Myc–His was generated by PCR amplification of full-length prohibitin (isoform 1; NCBI accession number NM_001281496.1) and cloning the product into the EcoRI–BamHI sites of the pcDNA.3.1/Myc-His(–) A plasmid (Invitrogen). The construct encoding DRP1–GFP was generated by PCR amplification of full-length *Drp1* (isoform 3; NCBI accession number NM_005690.4) and cloning the product into the EcoRI–BamHI sites of the pEGFP-C1 plasmid (Clontech). The construct encoding MFF–GFP was generated by PCR amplification of full-length *MFF* (isoform a; NCBI accession number NM_001277061.1) and cloning the product into the XhoI–BamHI sites of the pEGFP-C1 plasmid (Clontech). The construct encoding FIS1–GFP was generated by PCR amplification of full-length *Fis1* (NCBI accession number NM_016068.2) and cloning the product into the EcoRI–BamHI sites of the pEGFP-C1 plasmid (Clontech). The construct encoding MiD49–GFP was generated by PCR amplification of full-length *MiD49* (isoform 1; NCBI accession number NM_139162.3) and cloning the product into the EcoRI–BamHI sites of the pEGFP-C1 plasmid (Clontech). The construct encoding MiD51–GFP was generated by PCR amplification of full-length *MiD51* (isoform 1; NCBI accession number 2.NM_019008.5) and cloning the product into the EcoRI–BamHI sites of the pEGFP-C1 plasmid (Clontech). The construct encoding mtPAGFP was provided by Addgene. The siRNAs were synthesized by GenePharma, and the sequences used were as follows: siCTRP1 against the target sequence 5′-CCGGCAAGTTCTACTGCTAtt-3′, siDRP1 against the target sequence 5′-AACGCAGAGCAGCGGAAAGAGtt-3′, siMFF against the target sequence 5′- AACGCTGACCTGGAACAAGGAtt-3′, siMiD49 against the target sequence 5′-GCAGTATGAGCGTGACAAAAtt-3′, siMiD51 against the target sequence 5′-GATTGACGACATTGGCUATtt-3′, siFis1 against the target sequence 5′-GTACAATGATGACATCCGTAAtt-3′, siBak against the target sequence 5′- AATGCCTATGAGTACTTCACCtt-3′, and siBax against the target sequence 5′-TATGGAGCTGCAGAGGATGtt-3′. As a control, Silencer Negative Control siRNA (GenePharma) was used.

### Mammalian cell growth, transfection and drug treatment

Primary MEFs were isolated from wild-type and *Ctrp1*^fl/fl^ embryos. Primary MEFs, HeLa cells, 293T cells and U2OS cells were maintained in Dulbecco’s modified Eagle’s medium (DMEM; Invitrogen) supplemented with 10% FBS (Invitrogen), 100 U/ml penicillin and 100 μg/ml streptomycin (Invitrogen). For inactivation of endogenous *Ctrp1*, *Ctrp1*^fl/fl^ MEFs were infected with a Cre-expressing (Puro) lentivirus (Genetarget) at a dose of 1 × 10^7^ plaque-forming units and cultured for 24 h in DMEM supplemented with 10% FBS and 3 μg/ml puromycin (Sigma). Cells (2 × 10^5^ cells/well) were seeded in a six-well plate for 18 h prior to transfection. Plasmid transfections were performed in Opti-MEM medium (Invitrogen) with 3 μl of Lipofectamine 2000 (Invitrogen) for 4 h. The cells were then seeded onto microscope dishes with cover-glass bottoms (SPL) at a concentration of ~0.5 × 10^5^ cells/ml. For RNAi transfections, cells were seeded onto six-well plates at 30–40% density (or an equivalent density). Then, 4 μl of Lipofectamine 2000 (Invitrogen) and 40 nM RNAi oligonucleotides were added to each well. Cells were retransfected 24 h later with 3 μl of Lipofectamine 2000 and the appropriate plasmid DNA. For mitochondrial staining, cells were treated with 100 nM MitoTracker Red CMXRos (Invitrogen) in DMEM for 20 min prior to fixation. For staurosporine (STS; Sigma), actinomycin D (Sigma) or etoposide (Sigma) treatment, cells were seeded on poly-l-lysine-coated coverslips (0.1 mg/ml, Sigma) and incubated with 1 μM staurosporine for 4 h, 10 μM actinomycin D for 8 h or 20 μM etoposide for 16 h in serum-containing medium. Treatment with DMSO was used as the negative control.

### Electron microscopy

For preparation of samples for cellular transmission electron microscopy (TEM), MEFs (5.0 × 10^5^ cells/well) were seeded onto six-well plates for 24 h. The rabbit polyclonal antibody to CTRP1 was reacted with a 1:3 dilution of 5-nm gold-conjugated goat anti-rabbit IgG (Invitrogen) or 10-nm gold-conjugated goat anti-rabbit IgG (Sigma) in DMEM for 1 h at 37 °C. The cells were then rinsed with PBS (pH 7.4, 1 mM), and gold-conjugated polyclonal anti-CTRP1 was added to DMEM supplemented with 10% FBS. After 6 h of incubation at 37 °C, the cells were washed three times with PBS, centrifuged and then washed three times with blocking buffer (0.03% bovine serum albumin and 0.01% NaN_3_ in PBS). The samples were then fixed with 2% glutaraldehyde paraformaldehyde in 0.1 M PBS (pH 7.4) for 2 h and washed three times for 30 min in 0.1 M PBS (pH 7.4, 1 mM). The cells were then postfixed with 1% OsO_4_ (osmium tetroxide) dissolved in 0.1 M PBS (pH 7.4) for 2 h, dehydrated in an ascending gradual series (50%, 60%, 70%, 80%, 90%, 95% and 100%) of ethanol and infiltrated with propylene oxide. Specimens were embedded using a Poly/Bed 812 kit (Polysciences, USA). After the samples were embedded in pure, fresh resin at 60 °C in an electron microscope oven (TD-700, Dosaka, Japan) for 24 h, 300-nm-thick sections were initially cut and stained with toluidine blue for observation under a light microscope (Olympus BX40, Japan). Then, 80-nm-thick sections were double-stained with 7% uranyl acetate and lead citrate for contrast staining (20 min). The sections were then cut using a Leica Ultracut UCT Ultramicrotome (Leica Microsystems, Austria). All samples were observed using TEM (JEM-1011, JEOL, Japan) at an acceleration voltage of 80 kV.

### Subcellular fractionation

As previously described^33,34^, MEFs or U2OS cells were washed with PBS (Ca^2+^- and Mg^2+^-free), isolated, washed, resuspended in isolation buffer (IB; 50 mM Tris pH 7.4, 150 mM NaCl, 1 mM EGTA and 250 mM sucrose) and homogenized in a glass Dounce homogenizer with 30 strokes of a Teflon pestle. The homogenate was spun twice at 800 × *g* for 10 min to remove entire cells and nuclei, and the supernatant was centrifuged twice for 10 min at 8000 × *g*. The resulting pellet (crude mitochondrial fraction) was collected, and the supernatant was centrifuged for an additional 30 min at 100,000 × *g*. The resulting pellet (LM fraction) and supernatant (cytosolic fraction) were centrifuged again at 100,000 × *g* to further purify the fractions. The crude mitochondrial fraction was further purified by centrifugation twice at 8000 × *g* for 10 min. The obtained pellet was purified by centrifugation at 95,000 × *g* for 30 min on a 30% Percoll density gradient in IB. The low-density band was centrifuged for 10 min at 7000 × *g*, and the supernatant was centrifuged for 1 h at 100,000 × *g* to obtain the MAM pellet. The high-density band was centrifuged twice for 10 min at 7000 × *g*, and the resulting pellet contained the mitochondria. The obtained mitochondrial layer was washed free of Percoll and resuspended in IB. Isolation of mitochondria from MEF or U2OS cell lysates was performed with a Mitochondria Isolation Kit for Cultured Cells according to the manufacturer’s protocol (Thermo Fisher Scientific, Inc.).

### Membrane fractionation assay and trypsin-protection assay

For the membrane fractionation assay, the ER was isolated and resuspended in IB or IB containing 0.1 M Na_2_CO_3_ or 1% Triton X-100. After 15 min of incubation on ice, the samples were centrifuged at 20,000 × *g* for 30 min to separate the pellet and soluble fractions. For the trypsin protection assay, the ER was incubated with 10 μg/ml trypsin (Promega) in the absence of protease inhibitors, and the reaction was terminated by the addition of PMSF to a final concentration of 2 mM.

### Immunoprecipitation and western blotting

For immunoprecipitation experiments, nontransfected and transfected cells were lysed in whole-cell extraction buffer (10 mM HEPES (pH 7.9), 400 mM NaCl, 0.1 mM EDTA, 5% glycerol, 1 mM DTT and protease inhibitors). Nontreated cell lysate or anti-CTRP1 (1 μg) was added to the lysate, followed by the addition of anti-Flag-tagged agarose (Sigma) or protein A–agarose (Upstate Biotech) in TEG reaction buffer (20 mM Tris–HCl at pH 7.4, 1 mM EDTA, 10% glycerol, 1 mM DTT and 150 mM NaCl), and the mixture was stirred for 3 h or overnight at 4 °C. Immunoprecipitates were washed in TEG washing buffer (TEG reaction buffer containing 0.1% Triton-X 100). For Western blotting, cells were lysed in whole-cell extract buffer or homogenized using a MICCRA D-8 homogenizer (ART-moderne Labortechnik) in protein extraction buffer (20 mM HEPES (pH 7.9), 300 mM NaCl, 10 mM EDTA, 0.1% NP40, 100 mM KCl and protease inhibitors). Total protein was fractionated on a sodium dodecyl sulfate polyacrylamide gel and transferred to nitrocellulose membranes (Amersham Biosciences). Primary antibodies against the following proteins were used: DRP1, cytochrome C, FAS (BD Biosciences), phospho-Drp1-Ser616 (Cell Signaling) BIP, calreticulin, calnexin, kinectin1, prohibitin, TOM20, PARP, ACSL4 (Santa Cruz Biotechnology), tubulin, calbindin, Flag (Sigma), CTRP1, Myc, cytochrome C, COX4 (Abcam) and caspase-3 (Cell Signaling). The secondary antibodies were fluorescein-conjugated anti-rabbit IgG, anti-mouse IgG, anti-goat IgG (Invitrogen) and HRP-conjugated antibodies to rabbit, mouse or goat antibody (Zymed Laboratories). Protein–antibody complexes were detected with the ECL Plus system (Amersham Biosciences).

### Live-imaging and confocal microscopy

Live imaging of cells was performed with a spinning disc confocal system (A1C; Nikon). For live cells, imaging was performed at 37 °C and 5% CO_2_ in an LCI chamber (Chamlide TC; LCI), and images were taken with a ×60 numerical aperture (NA) 1.4 oil objective lens. For fixed cells, imaging was performed with a laser-scanning confocal microscope (LSM780; Carl Zeiss). Images were taken with a ×63 oil objective lens (NA 1.4). Images were analyzed using Carl Zeiss Elements, Photoshop (Adobe), IMARIS (Bitplane AG) or ImageJ (US National Institutes of Health; NIH) software. Scale bars were generated using Carl Zeiss Elements and ImageJ.

### Mitochondrial length and DRP1 fluorescence analysis

For measurement of mitochondrial length, the *z*-series stacks were imaged in increments of 0.2 μm centered on the middle focal plane of the mitochondria. We analyzed the images of flat regions of cells with clearly compartmentalized mitochondria, and 20–30 mitochondria per cell were measured using Carl Zeiss Elements software. For analysis of DRP1 fluorescence, only mitochondria-associated DRP1 puncta on consecutive *z*-planes were quantified with ImageJ software (NIH). Statistical analyses were performed using SigmaPlot (Systat Software, Inc.). Data are presented as the mean and s.e.m. or s.d. of at least three experiments.

### Immunofluorescence and histology

Cultured cells were fixed in 4% formaldehyde in PBS for 20 min at room temperature and, after being washed with PBS, were permeabilized with 0.1% Triton X-100 for 15 min. Cells were washed with PBS, blocked with 3% BSA in PBS for 1 h and incubated with primary antibodies in PBS for ~1–3 h at room temperature. After being washed with PBS, the cells were incubated with secondary antibodies for 1 h at room temperature. The cells were then counterstained with 10 μM 4,6-diamidino-2-phenylindole (DAPI) and mounted with Vectashield (VECTOR). Cryosections and paraffin-embedded Section (5-µm thick) of the brain and liver were fixed in 10% buffered formaldehyde and used for H&E staining, TUNEL assays, Nissl staining and immunofluorescence analyses.

### FAS-induced apoptosis

Twelve-week-old *Alb-Cre;Ctrp1*^fl/fl^ and *Ctrp1*^fl/fl^ male mice were injected via the tail vein with an activating FAS-specific antibody (clone JO2; 0.25 µg/g body weight (µg/g) in sterile saline solution; BD Pharmingen) or with a sterile saline solution as a control^35^. After 24 h, the indicated animals were euthanized for the analysis of apoptosis in the liver. Liver samples were fixed in 10% formalin for 24 h, embedded in paraffin or optimal cutting temperature compound and sectioned to a 5-µm thickness. Cryosections and paraffin-embedded sections were processed for H&E staining, TUNEL assays and immunofluorescence analysis. For measurement of the plasma AST and ALT levels, fresh blood was collected from 4-h-fasted mice and centrifuged at 1500 × *g* for 20 min. The resulting supernatant (serum) was used to measure AST and ALT levels using a Hitachi autoanalyzer.

### Statistical analysis

Differences between two experimental groups were analyzed using Student’s *t*-test or the Mann–Whitney *U* test. To compare three or more groups, we used one-way ANOVA with Šidák’s correction. *P* < 0.05 was considered statistically significant.

## Results

### CTRP1 is an ER membrane protein

**Fig. 1:**
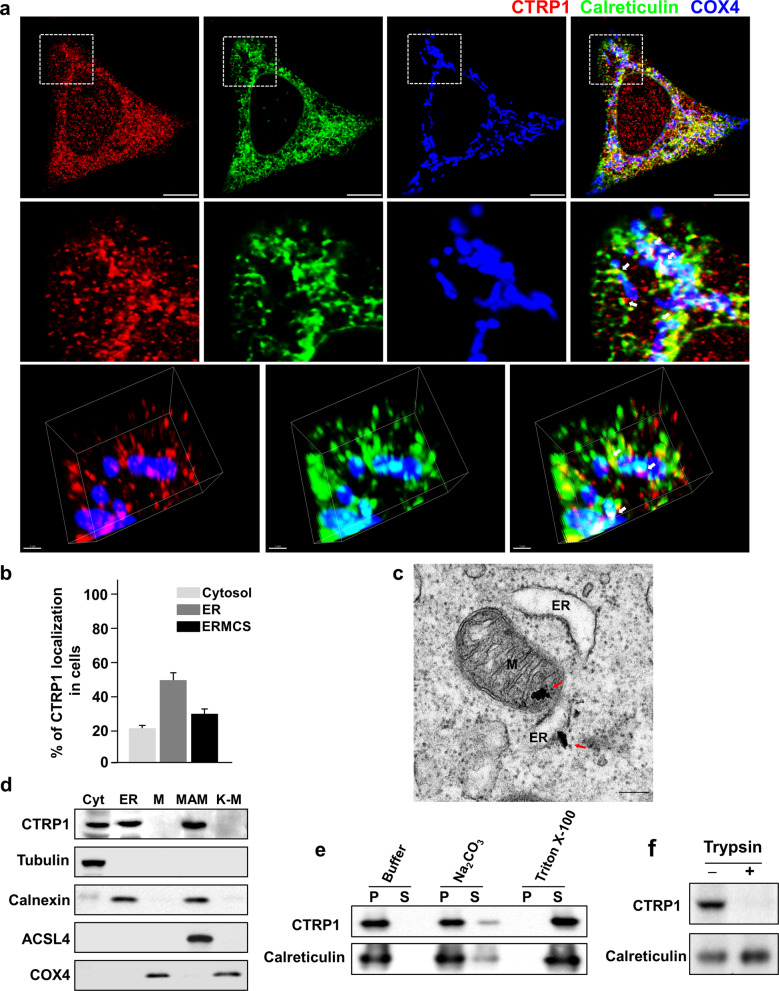
CTRP1 is an ER membrane protein. **a** Representative low-magnification images (top), high-magnification images of the boxed areas (middle) and 3D reconstruction images (bottom) of mouse embryonic fibroblasts (MEFs) immunostained with CTRP1 (red), the ER marker calreticulin (green), and the mitochondrial protein COX4 (blue) antibodies. The white arrows indicate CTRP1 in the EMCSs. Scale bars, 10 µm (top) and 1 µm (bottom). **b** Quantitation of CTRP1 localization to the cytosol, ER and ERMCSs in MEFs. **c** Representative electron micrograph of MEFs stained with anti-CTRP1 conjugated to 10-nm gold nanoparticles (red arrows). M mitochondrion, ER endoplasmic reticulum. Scale bar, 200 nm. **d** Western blot analysis of subcellular fractions of MEFs. Cyt cytosol, MAM mitochondria-associated ER membrane, K-M Kit-purified mitochondria. The subcellular fractions were immunoblotted with antibodies against the cytosolic marker tubulin, the ER marker calnexin, the MAM marker ACSL4, and the mitochondrial marker COX4. **e** Western blot analysis of the pellet (P) and supernatant (S) fractions collected after ER samples were incubated with buffer, Na_2_CO_3_ or Triton X-100. **f** Western blot analysis of ER fractions that were treated with or without trypsin. Throughout, the data are presented as the mean ± s.e.m.

We first examined the subcellular localization and dynamic distribution of CTRP1 in various mouse and human cell lines. CTRP1 was distinctively localized to the ER, EMCSs and cytosol (Fig. 1a, b and Supplementary Fig. 1a–g). Further analyses using ectopic expression of GFP-tagged CTRP1 showed that GFP–CTRP1 localized to the ER and to mitochondrial fission sites in living cells (Supplementary Fig. 1f). Electron microscopic (EM) analysis revealed that immunogold-conjugated CTRP1 localized to the mitochondria-associated ER membrane (MAM) (Fig. 1b and Supplementary Fig. 2). A previous paper suggested that CTRP1 has an N-terminal signal peptide^19^, and our sequencing analysis of this N-terminal signal peptide revealed that it contains a putative transmembrane domain (Supplementary Fig. 3a). To verify the function of this transmembrane domain, we expressed truncated versions of Myc-tagged CTRP1 in HeLa cells and examined their localization patterns (Supplementary Fig. 3c, d). We found that the CTRP1 mutants lacking the N-terminal domain localized to the cytosol, whereas those lacking the C-terminal C1q domain localized to the ER (Supplementary Fig. 3b and d). To further confirm the functionality of the N-terminal transmembrane domain of CTRP1, we substituted the cysteine residue at position 13 of this domain with either alanine (CTRP1^C13A^) or arginine (CTRP1^C13R^). Our results revealed that wild-type CTRP1 (CTRP1^WT^) and CTRP1^C13A^ localized to the ER, whereas CTRP1^C13R^ localized to the cytosol (Supplementary Fig. 3e). A fractionation assay using mouse embryonic fibroblasts (MEFs) revealed that CTRP1 was present in the cytosolic, ER and MAM fractions but not in the mitochondrial fraction (Fig. 1c). To further test whether CTRP1 is an ER membrane- or ER lumen-associated protein, we exposed ER fractions to high-pH or detergent conditions and then further collected the pellet and supernatant fractions. We found that CTRP1 remained associated with the membrane at high pH but was partitioned into the soluble fraction in the presence of detergent (Fig. 1d). In addition, trypsin protection assays performed on the purified ER fraction revealed that CTRP1 was sensitive to trypsin treatment, whereas the ER lumen protein calreticulin was not (Fig. 1e, f). Taken together, these results indicate that CTRP1 is an ER membrane-bound protein that contains an N-terminal transmembrane domain.

### CTRP1 regulates mitochondrial morphology

**Fig. 2:**
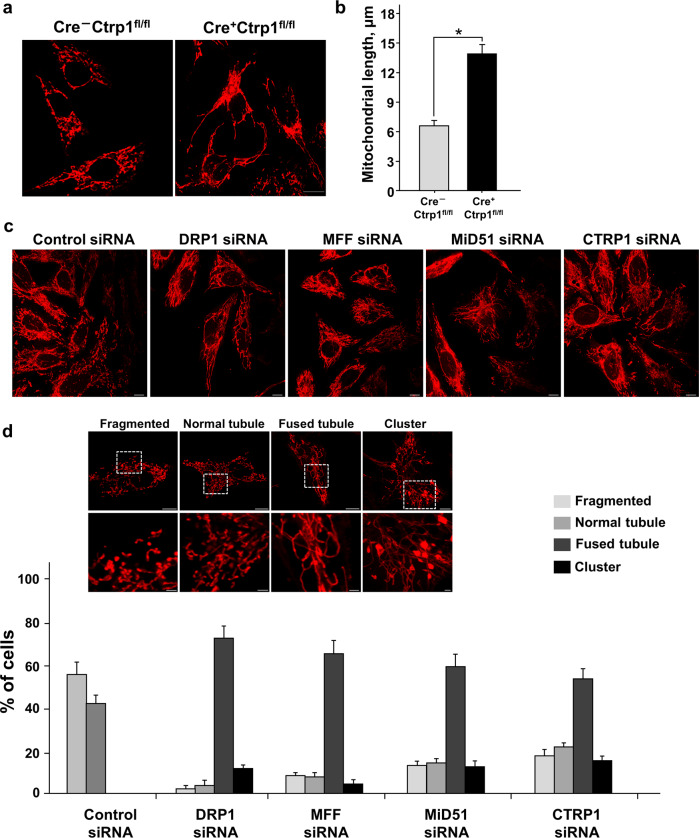
CTRP1 regulates mitochondrial morphology. **a** MitoTracker-stained Cre^–^Ctrp1^fl/fl^ and Cre^+^Ctrp1^fl/fl^ MEFs. **b** Quantification of mitochondrial lengths in Cre^–^Ctrp1 ^fl/fl^ and Cre^+^Ctrp1 ^fl/fl^ MEFs (*n* = 256–427 mitochondria of 30–50 cells). **c** and **d** MitoTracker staining (**c**) and quantification of mitochondrial morphology (**d**) in human bone osteosarcoma (U2OS) cells transfected with the indicated siRNAs (*n* = 300 cells). Quantitative data were obtained by analysis of random samples and are presented as the mean ± s.e.m. of three independent experiments *P < 0.001. Scale bars, 10 μm in (**a**) and (**c**), 10 µm (top) and 2 µm (bottom) in (**d**).

To study the precise role of CTRP1 in specific cells and tissues, we generated conditional knockout mice in which exon 3 of *Ctrp1* was deleted using the Cre/*lox*P system (Supplementary Fig. 4). We first transfected embryos harboring *loxP*-flanked (floxed) *Ctrp1* (*Ctrp1*^fl/fl^) with a lentivirus encoding the Cre recombinase and collected MEFs for analysis (Supplementary Fig. 4a–d). The Cre^+^*Ctrp1*^fl/fl^ (*Ctrp1*^–/–^) MEFs showed elongated mitochondria (Fig. 2a and Supplementary Fig. 5a), which are a characteristic sign of a fission defect, but did not show any morphological change in the ER or Golgi apparatus (Supplementary Fig. 6). The mitochondrial length was significantly greater in the Cre^+^*Ctrp1*^fl/fl^ MEFs than in the Cre^–^*Ctrp1*^fl/fl^ MEFs that did not express Cre (*Ctrp1*^+/+^ MEFs) (Fig. 2b). Next, we used siRNAs to deplete DRP1, MFF, Mid51 or CTRP1 from human bone osteosarcoma (U2OS) cells (Supplementary Fig. 5c). DRP1 is critical to the induction of mitochondrial fission, and its adaptors, MFF and MiD51, regulate mitochondrial fission by recruiting DRP1 in mitochondria^23–26^. Consistent with our observations in the Cre^+^*Ctrp1*^fl/fl^ MEFs, we found that depletion of CTRP1 from U2OS cells significantly altered the mitochondrial morphology, similar to mitochondrial morphology defects following depletion of DRP1, MFF or MIEF1 (also known as MiD51) from U2OS cells (Fig. 2c, d and Supplementary Fig. 5b). The mitochondrial morphology defects in the U2OS cells treated with a CTRP1-specific siRNA (siCTRP1) were substantially rescued by overexpression of CTRP1 (Supplementary Fig. 7). We also analyzed the mitochondrial dynamics in the HeLa cells cotranfected with mito-RFP and mito-PAGFP after either control or CTRP1 siRNA treatment and found that CTRP1 was not associated with mitochondrial fusion (Supplementary Fig. 8). These results suggest that CTRP1 is involved in mitochondrial fission.

### CTRP1 interacts with DRP1

**Fig. 3:**
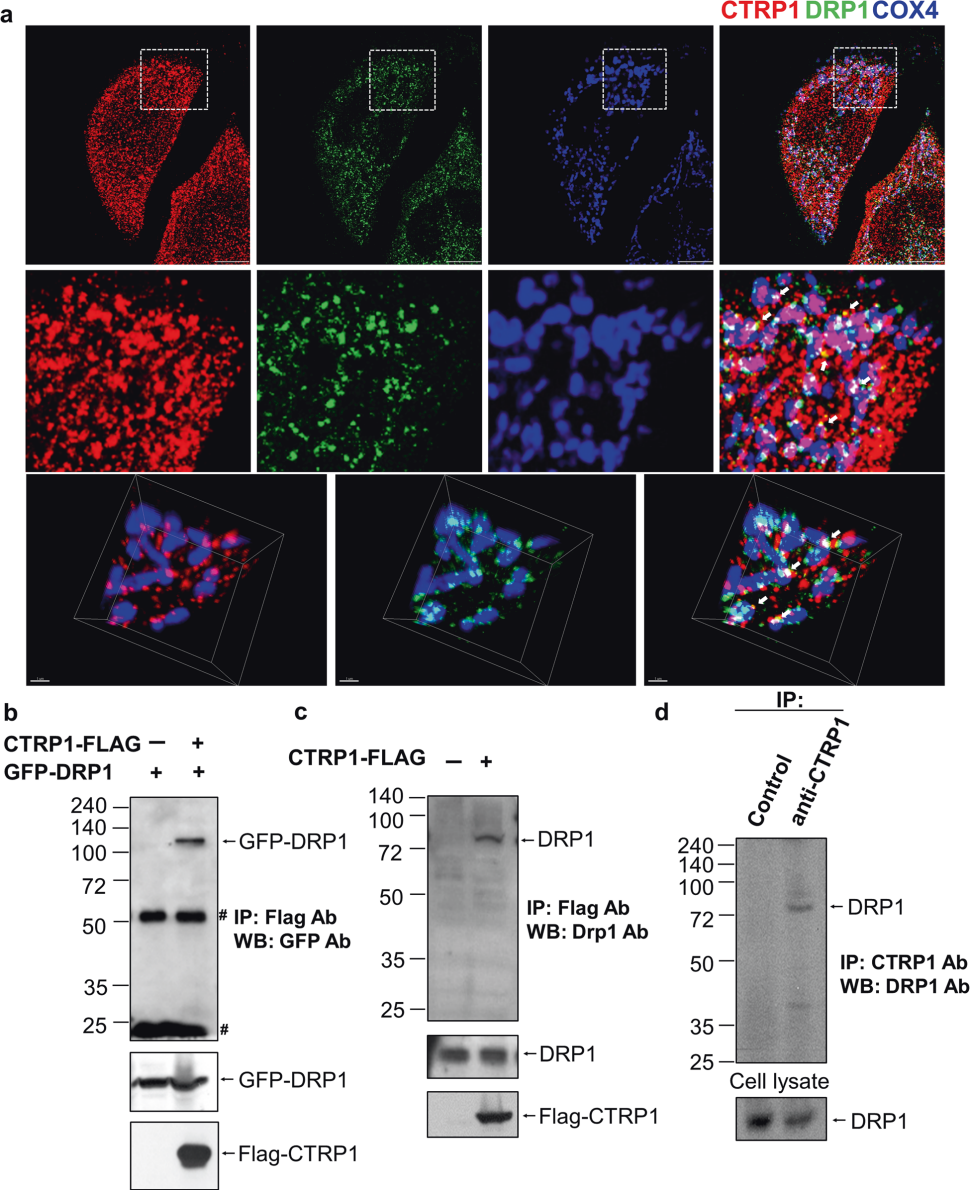
CTRP1 interacts with DRP1. **a** Representative low-magnification images (top), high-magnification images of the boxed areas (middle) and 3D reconstruction images (bottom) of MEFs immunostained with antibodies against CTRP1 (red) and DRP1 (green) and the mitochondrial protein COX4 (blue). The white arrows indicate colocalization of CTRP1 and DRP1. Scale bars, 1 µm (top) and 10 µm (bottom). **b** Whole-cell lysates (WCLs) of 293T cells cotransfected with constructs expressing Flag–CTRP1 and GFP–DRP1 were immunoprecipitated (IP) with anti-Flag beads and then immunoblotted (IB) with a GFP-specific antibody. Asterisks indicate immunoglobulin G (IgG). **c** Interaction between endogenous DRP1 and Flag-CTRP1; 293T cells were transfected with Flag–vector (Lane 1) or Flag–CTRP1 (Lane 2), and WCLs were immunoprecipitated with anti-Flag beads and blotted with anti-DRP1. **d** Interaction between endogenous DRP1 and CTRP1. WCLs of 293T cells were immunoprecipitated with anti-CTRP1 and blotted with anti-Drp1.

To investigate the function of ER-bound CTRP1 in mitochondrial fission, we first analyzed the subcellular localizations of Ctrp1 and Drp1 in MEFs and found that endogenous Ctrp1 partially colocalized with Drp1 at the EMCSs (Fig. 3a and Supplementary Fig. 1g). Next, we used immunoprecipitation (IP) assays to test for a physical interaction between Flag–CTRP1 and GFP–DRP1, between Flag–CTRP1 and endogenous DRP1 or between endogenous CTRP1 and DRP1. Our results confirmed that CTRP1 binds to DRP1 (Fig. 3b–d). To determine which CTRP1 domains interact with DRP1, we examined the physical interaction between Flag–CTRP1 truncated mutants and GFP–DRP1. We found that the C1q domain of CTRP1 interacts with DRP1 (Supplementary Fig. 9a). This finding suggests that the interaction between ER-bound CTRP1 and mitochondrial DRP1 may be required for mitochondrial fission events in the EMCSs. We also observed partial colocalization and physical interaction of CTRP1 with the mitochondrial DRP1 adaptor proteins MIEF2 (also known as MiD49) and MiD51 (Supplementary Fig. 9b, c). Additionally, we found that Mid49 or Mid51 did not affect the physical interaction between Flag–CTRP1 and GFP–DRP1 (Supplementary Fig. 9d). These findings suggest that the ER–mitochondrion interface might be necessary for mitochondrial fission. Moreover, our results collectively indicate that CTRP1 is an ER membrane protein that interacts with DRP1 at the EMCSs.

### CTRP1 recruits DRP1 and acts as a DRP1 adaptor

**Fig. 4:**
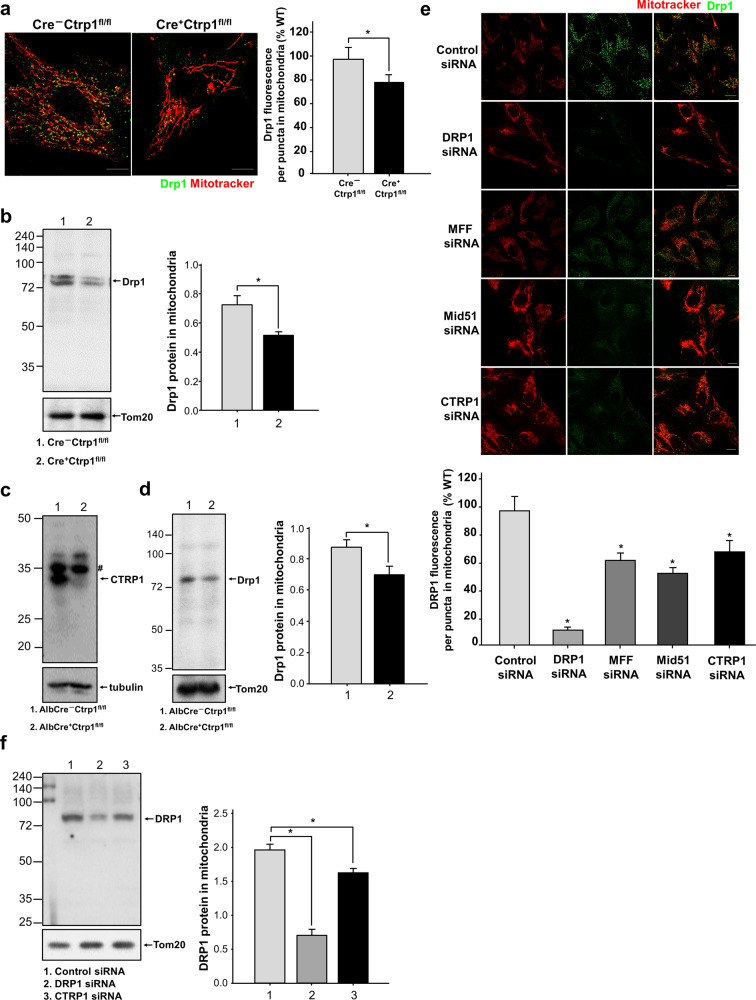
CTRP1 regulates the recruitment of DRP1 during mitochondrial fission. **a** and **e** MitoTracker and anti-Drp1 staining of the indicated cells. Quantification of the Drp1 fluorescence signal from three independent biological replicates is shown. **b**, **d** and **f** Immunoblotting of mitochondrial fractions and WCLs with antibodies against DRP1 and the mitochondrial protein Tom20. **c** Immunoblotting of the *Alb*–*Cre*^−^*Ctrp1*^*fl/fl*^ and *Alb*–*Cre*^+^*Ctrp1*^*fl/fl*^ cells with antibodies against CTRP1 and the cytosolic marker tubulin. The asterisks in **f** represent nonspecific bands. Data are presented as the mean and s.e.m. *P < 0.001. Scale bars, 10 μm (**a**) and (**e**).

As DRP1-interacting mitochondrial proteins influence the recruitment of DRP1 in mitochondria, we next analyzed the effects of CTRP1 on DRP1 recruitment to determine the role of CTRP1 in mitochondrial fission. The fluorescence intensity of the Drp1 signal was decreased in the mitochondria of the Cre^+^*Ctrp1*^fl/fl^ MEFs relative to those in the Cre^–^*Ctrp1*^fl/fl^ MEFs (Fig. 4a). To investigate whether Ctrp1 deficiency affects the Drp1 protein levels in mitochondria, we examined the protein levels of Drp1 and found that they were decreased in the mitochondria of the Cre^+^*Ctrp1*^fl/fl^ MEFs (Fig. 4b). We generated *Alb*–*Cre*;*Ctrp1*^fl/fl^ mice and confirmed that Ctrp1 was abolished in the *Alb*–*Cre*;*Ctrp1*^fl/fl^ liver cells (Fig. 4c) and further found that Drp1 protein levels were decreased in the mitochondria of the *Alb*–*Cre*;*Ctrp1*^fl/fl^ liver cells (Fig. 4d). Consistent with these findings, depletion of CTRP1 in U2OS cells significantly altered the mitochondrial localization of the DRP1 signal, whereas depletion of MFF or MiD51 had no such effect (Fig. 4e). CTRP1 depletion also resulted in a significant decrease in the DRP1 protein levels (Fig. 4f), indicating that CTRP1 is involved in the recruitment of DRP1. However, CTRP1 did not alter the phospho-Drp1 levels (Supplementary Fig. 9e). Collectively, these results suggest that ER-bound CTRP1 interacts with DRP1, acts as a DRP1 adaptor and regulates mitochondrial fission events via its effects on DRP1 recruitment.

### Ablation of CTRP1 leads to apoptotic resistance

**Fig. 5:**
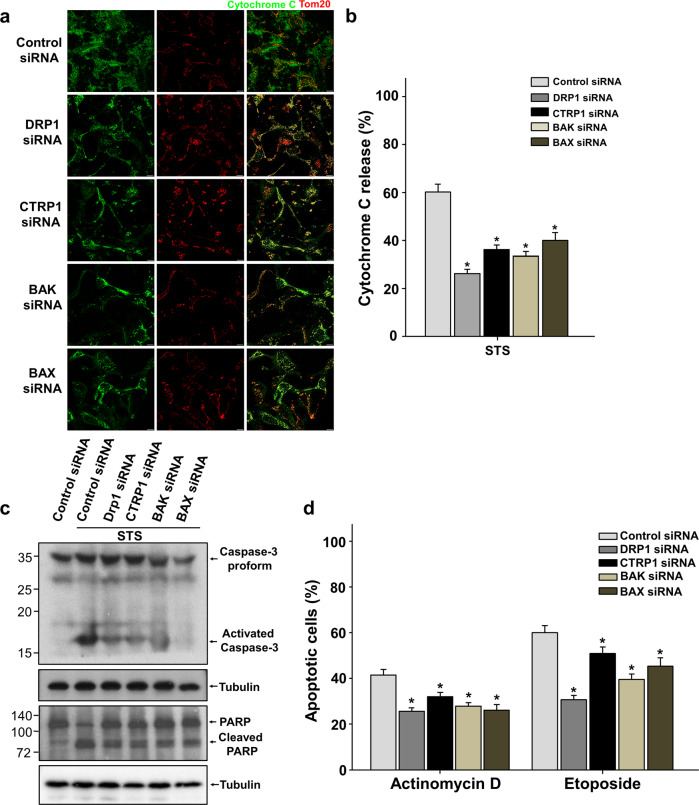
CTRP1-deficient cells are less sensitive to the induction of apoptosis. **a–c** Apoptotic analysis of U2OS cells transfected with the indicated siRNAs and treated with STS, MitoTracker (red) and anti-cytochrome C antibody (green) staining (**a**), quantification of cytochrome C release (*n* = 600 cells) (**b**) and immunoblotting with caspase-3, PARP and the cytosolic marker tubulin antibodies (**c**). **d** Quantification of fragmented nuclei in cells treated with actinomycin D or etoposide (*n* = 600 cells). Data are presented as the mean and s.e.m. Scale bars, 10 μm in (**a**).

DRP1 influences the oligomerization of the proapoptotic protein BAX through mitochondrial membrane remodeling and is thereby involved in apoptotic resistance^30^. As mammalian cells undergo apoptosis, many proteins, including cytochrome c in the mitochondrial intermembrane space, are forced out from the mitochondria when the permeability of the mitochondrial outer membrane (MOM) is increased via pores formed by the proapoptotic BCL-2 family members BAX and BAK^31,32^. Depletion of BAX or BAK is associated with apoptotic resistance, and the mitochondrial DRP1 adaptor proteins that regulate the recruitment and oligomerization of DRP1 have also been implicated in resistance to cell death^25,26^. Accordingly, we next investigated the potential function of CTRP1 in apoptosis by examining whether CTRP1 depletion affected the cell death induced by staurosporine (STS), actinomycin D or etoposide. Normal U2OS cells had filamentous mitochondria that maintained their cytochrome c under normal conditions, whereas the STS-treated U2OS cells showed fragmented mitochondria and the release of cytochrome c into the cytosol (Supplementary Fig. 10a, b). Activated caspase-3 (Supplementary Fig. 10c) and caspase-3-mediated cleavage of PARP (Supplementary Fig. 10d) were detected in the STS-treated U2OS cells. To elucidate the potential function of CTRP1 in apoptotic resistance, we examined cytochrome c release in the siCTRP1-treated U2OS cells. Compared to the control siRNA-treated U2OS cells, the CTRP1-depleted cells exhibited decreased cytochrome c release similar to that observed in the DRP1-, BAX- and BAK-ablated cells (Fig. 5a, b). Caspase-3 activation and PARP cleavage were also decreased in the CTRP1-depleted cells (Fig. 5c). Finally, we analyzed whether CTRP1 depletion affected the survival of cells treated with actinomycin D or etoposide. Indeed, our quantification analyses showed that the number of apoptotic cells was decreased in the CTRP1-ablated cell cultures treated with these agents (Fig. 5d). Apoptotic resistance, such as caspase-3 activation, PARP cleavage, cytochrome c release and apoptotic HeLa cells after treatment with a CTRP1-specific siRNA (siCTRP1), was generally rescued by overexpression of CTRP1 (Supplementary Fig. 10e–g). Our results therefore suggest that CTRP1 plays an important role in the ability of cells to resist apoptosis.

### Ctrp1 depletion contributes to Fas-induced apoptotic resistance in liver cells and degeneration of neuronal cells

**Fig. 6:**
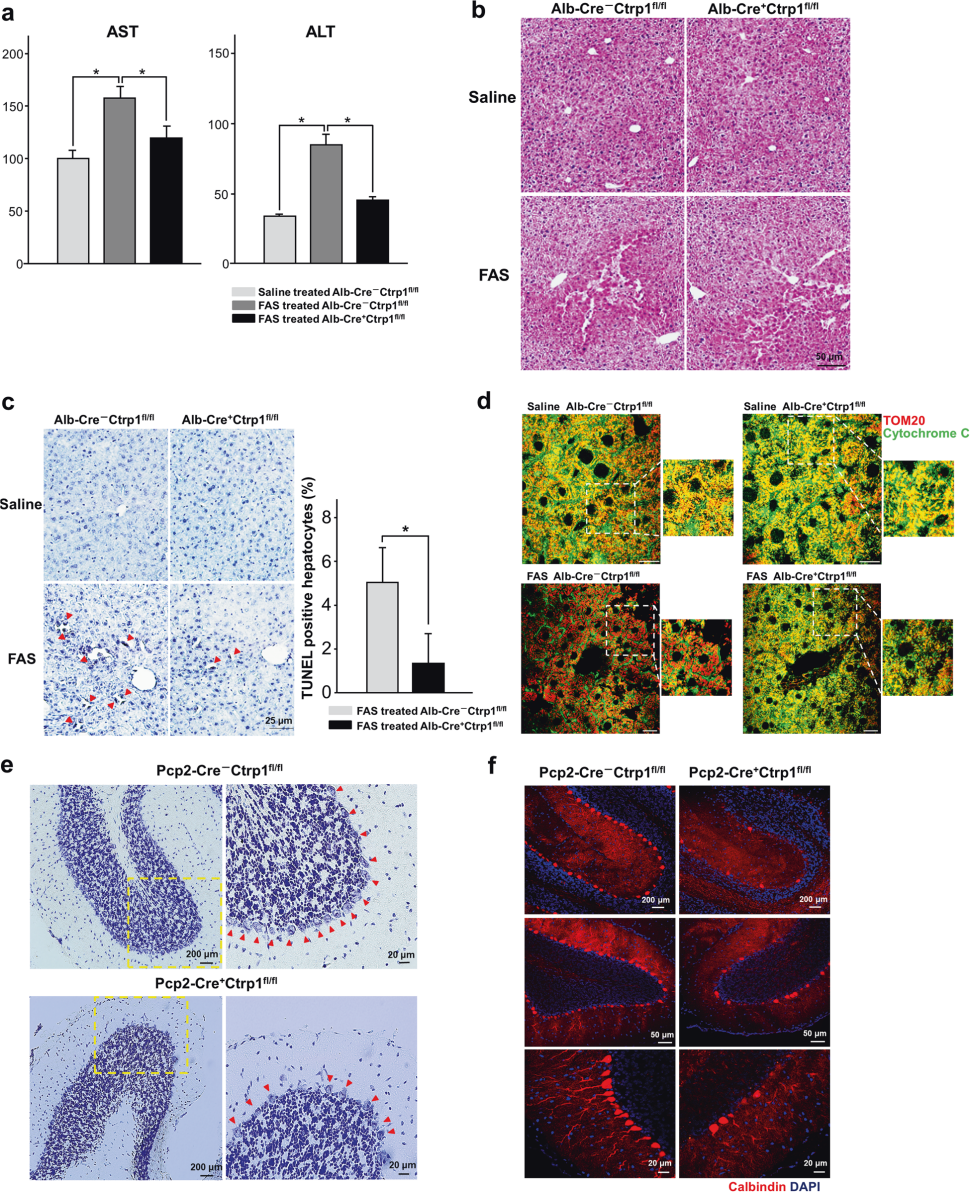
Ctrp1 deficiency causes resistance to Fas-induced apoptosis and triggers neuronal defects. **a**–**d** Plasma AST and ALT levels (**a**), hematoxylin and eosin (H&E) staining (**b**), TUNEL staining (**c**) and immunostaining with antibodies against cytochrome C (green) and Tom20 (red) (**d**) in liver sections obtained 24 h after 3-month-old mice were injected with anti-Fas (Fas) or saline, as indicated. **e** and **f** Nissl staining (**e**) and immunostaining for the Purkinje cell (PC) marker calbindin (**f**) in the cerebella of 10-week-old mice injected as indicated. Data are presented as the mean and s.e.m. Scale bars are as indicated.

FAS-induced cell death in the liver is known to depend on mitochondria^36,37^. To investigate whether Ctrp1 contributes to regulating apoptosis in vivo, we induced apoptotic liver damage in the *Alb*–*Cre*;*Ctrp1*^fl/fl^ and control *Ctrp1*^fl/fl^ mice by tail vein injection of FAS-specific antibodies. We then examined the serum levels of aspartate transaminase (AST) and alanine transaminase (ALT), which are indicators of liver damage, at 24 h post-injection. The anti-FAS-treated *Alb*–*Cre*;*Ctrp1*^fl/fl^ mice exhibited significantly smaller increases in AST and ALT levels than the anti-FAS-treated control *Ctrp1*^fl/fl^ mice (Fig. 6a), and their livers showed a more intact lobular architecture and lower levels of hemorrhagic infiltrates than those of the control mice (Fig. 6b). TUNEL assays revealed that the livers of the anti-FAS-treated *Alb*–*Cre*;*Ctrp1*^fl/fl^ mice exhibited less apoptotic cell death than those of the anti-FAS-treated *Ctrp1*^fl/fl^ mice (Fig. 6c). An analysis of subcellular cytochrome c distribution showed that less cytochrome c was released from mitochondria in the livers of the anti-FAS-treated *Alb*–*Cre*;*Ctrp1*^fl/fl^ mice than in those of the anti-FAS-treated *Ctrp1*^fl/fl^ mice (Fig. 6d). These results collectively suggest that Ctrp1 plays a crucial role in the ability of hepatocytes to resist apoptosis in vivo. Finally, since alterations in the genes involved in mitochondrial fission and fusion lead to neuronal dysfunction, which can result in the degeneration of Purkinje cells (PCs) in the cerebellum^38,39^, we examined whether Ctrp1 deletion could trigger neuronal degeneration in *Pcp2*–*Cre*;*Ctrp1*^fl/fl^ mice, in which *Ctrp1* is specifically knocked out in PCs. Indeed, the number of cerebellar PCs in the *Pcp2*–*Cre*;*Ctrp1*^fl/fl^ mice was much lower than that in the *Ctrp1*^fl/fl^ mice (Fig. 6e, f), suggesting that Ctrp1 is required for neuronal cell survival.

## Discussion

We show that CTRP1 is a novel ER membrane protein that interacts with the mitochondrial fission-related proteins DRP1, MiD49 and MiD51 at the mitochondrial fission site (Supplementary Fig. 12). Mitochondrial fission is centrally regulated by Drp1, which oligomerizes around mitochondrial constriction sites and can drive mitochondrial fission. At a mitochondrial constriction site, the GTPase dynamin 1 (dnm1 in yeast) and Drp1 oligomerize into a helical ring that is much smaller than the diameter of the mitochondrion^40–44^. Thus, certain Drp1-associated factors may help Dnm1 and Drp1 drive mitochondrial constriction. If this is the case, such factors could be necessary for mitochondrial division. Our findings indicate that the interaction between ER-bound CTRP1 and Drp1 is required to regulate mitochondrial morphology in EMCSs. Additionally, our data suggest that ER-bound CTRP1 may regulate the recruitment of DRP1 by cooperating with MiD49 and MiD51, which are known DRP1 adaptors.

Previous studies have focused on the function of secreted CTRP1, which has been shown to regulate metabolic disease and cardiovascular function^1–13^. In the present study, we identified CTRP1 as an ER membrane protein that localizes to the EMCSs and regulates mitochondrial fission. Interestingly, immunoblot analysis of N-terminal or C-terminal GFP-tagged CTRP1 indicated that CTRP1 can have membrane-bound and secreted forms (Supplementary Fig. 11). The Peptide Cutter and PROSPER programs predicted that CTRP1 could have potential cleavage sites in its short variable domain (data not shown), adding weight to the idea that CTRP1 has both secreted and membrane-bound forms. Based on the existing evidence and our present findings, we speculate that CTRP1 could have a dual function, wherein its secreted form regulates metabolic and cardiovascular functions while its ER-bound form regulates mitochondrial fission. Given that mitochondrial dynamics are also critical for metabolic and cardiovascular functions^45^, it is necessary to further investigate whether the secreted form of CTRP1 can indirectly modulate mitochondrial dynamics.

ER tubules are wrapped around mitochondrial constriction sites and fission sites^27^. The ER–mitochondrion connection is mediated by the ER–mitochondria encounter structure (ERMES) complex in yeast and by the mitochondrial fusion protein mitofusin 2 (MFN2) in mammalian cells, both of which tether the ER to mitochondria in their respective cells^46,47^. Interestingly, depletion of the ERMES complex triggers a mitochondrial fission defect, whereas MFN2 deficiency leads to a mitochondrial fusion defect^39^. This finding raises the possibility that, reminiscent of the ERMES complex in yeast, the ER of mammalian cells may contain yet-unknown fission-associated factors beyond MFN2. Here, we provide multiple lines of evidence showing that ER-associated CTRP1 participates in mitochondrial fission events via an interaction with the mitochondrial fission protein DRP1.

The DRP1 adaptor proteins MFF, MiD49 and MiD51 form contacts with the ER in the EMCSs^27,48^. Recently, Osellame et al. investigated proteins that interact with MFF, MiD49 or MiD51 in the EMCSs^26^. These researchers showed that both MFF and MiD51 bound DRP1 but not mitochondrial Spire1 or the ER membrane proteins INF2 and Syn17, which are known to support mitochondrial constriction through actin polymerization^28,29,49^. This finding suggested that ER-bound proteins can interact with MFF, MiD49 and MiD51 or with DRP1 in the MAM. Interestingly, Ji et al. suggested that ER-bound MFF can mediate DRP1 oligomerization and that ER-associated DRP1 regulates mitochondrial fission^50^. Thus, further studies should investigate whether ER-bound CTRP1 contributes to DRP1 oligomerization. Here, we showed that CTRP1 is an ER-associated DRP1 adaptor that recruits DRP1 during mitochondrial fission. This recruitment of DRP1 could be necessary for the interaction with the mitochondrial DRP1 adaptors MFF, MiD49 and MiD51 and/or the ER-associated DRP1 adaptor CTRP1. Taken together, our findings and those in the literature suggest that complex networks of mitochondrial and ER-associated DRP1 adaptors critically contribute to mitochondrial fission by facilitating the recruitment and maturation of DRP1.

Studies have shown that during apoptosis, DRP1 affects Bax oligomerization via mitochondrial membrane remodeling^29^ and that ablation of the mitochondrial DRP1 adaptors MiD49 and MiD51 protects cells against apoptosis by modulating mitochondrial crista remodeling^25^. These results suggest that defective recruitment and/or assembly of DRP1 causes apoptotic resistance. Here, we showed that CTRP1 inactivation triggers resistance to FAS-induced apoptosis in liver cells. Thus, our results indicated that ER-associated CTRP1 can regulate apoptosis by recruiting DRP1 to the MAM. Finally, mitochondrial fission and fusion defects are known to cause neuronal degeneration, and a previous report showed that Drp1 deletion leads to neuronal degeneration in *Pcp2–Cre;Drp1*^*fl/fl*^ mice, in which *Drp1* is specifically knocked out in PCs^38,39^. We showed that the *Pcp2–Cre;Ctrp1*^*fl/fl*^ mice also exhibit neuronal degeneration of PCs. Our data thus collectively indicate that ER-associated CTRP1 contributes to neuronal survival and that the mitochondrial fission defects triggered by CTRP1 deficiency contribute to resistance to cell death and neuronal degeneration.

In conclusion, we herein demonstrated that CTRP1 acts as an ER membrane protein to regulate mitochondrial fission via an interaction with DRP1 and identified a potential mechanism wherein the recruitment of DRP1 by CTRP1 could possibly enhance Drp1 ring assembly. We also showed that CTRP1 deficiency induces apoptotic resistance and neuronal degeneration and thereby phenocopies DRP1 ablation. We thus propose a novel role for the cytosolic form of CTRP1. This work provides new insight into the etiology of human disorders associated with mitochondrial fission defects.

## Supplementary information

Supplementary Information
