# Supplementary material for: ER-associated CTRP1 regulates mitochondrial fission via interaction with DRP1
Source: Exp Mol Med. 2021 Nov 26;53(11):1769–80. doi: 10.1038/s12276-021-00701-z (PMC8639813; doi:10.1038/s12276-021-00701-z)
Supplement: Supplementary file 1 — Supplementary Information [file 12276_2021_701_MOESM1_ESM.pdf]

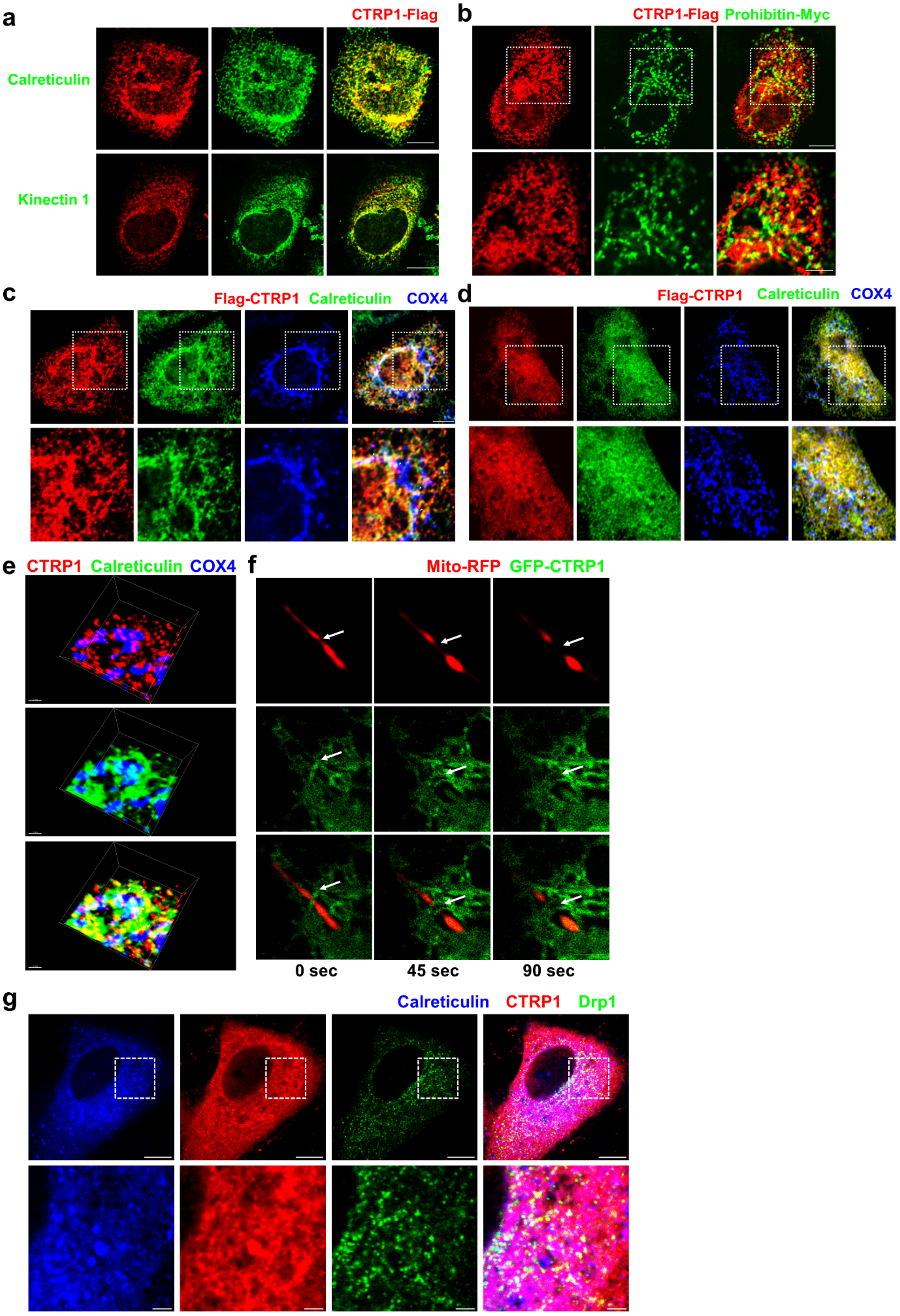
a
CTRP1-Flag
Calreticulin
Kinectin 1
b
CTRP1-Flag Prohibitin-Myc
c
Flag-CTRP1 Calreticulin COX4
d
Flag-CTRP1 Calreticulin COX4
e
CTRP1 Calreticulin COX4
f
Mito-RFP GFP-CTRP1
0 sec
45 sec
90 sec
g
Calreticulin CTRP1 Drp1

Supplementary Figure 1 CTRP1 partially localizes to the mitochondria, ER and mitochondrial fission sites.

(a-d) HeLa cells (a, b and c) and MEFs (d) transfected with the indicated plasmids were immunostained with antibodies against Flag, CTRP1, the ER marker, Kinectin 1 and calreticulin and the mitochondrial protein, prohibitin and COX4.

(e) HeLa cells were co-immunostained with the indicated antibodies.

(f) Time-lapse images of U2OS cells transfected with Mito-RFP and GFP-CTRP.

The white arrows in (f) indicate the location of CTRP1 in the EMCS as determined from panels

(g) Representative images of MEFs immunostained with CTRP1 (red), DRP1 (green) and the ER marker calreticulin (blue) antibodies.

(c to g). Scale bars, 10 μm (a-d), 1 μm (e) and 5 μm (f).

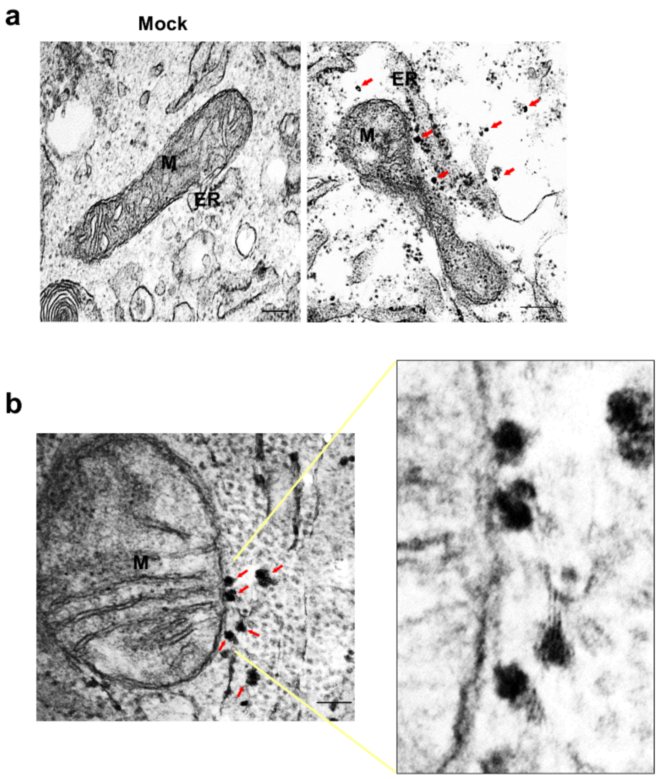

Supplementary Figure 2 CTRP1 localizes to the ER–mitochondrion contact site.

(a) An immunogold electron micrograph of MEFs exposed to anti-CTRP1 conjugated with 10-nm gold particles. (M: mitochondria, ER: endoplasmic reticulum)

(b) An immunogold electron micrograph of HeLa cells exposed to anti-CTRP1 conjugated with 5-nm gold particles. Scale bars, 200 nm in (a) 100 nm in (b). Red arrows indicate CTRP1 conjugated with gold particles.

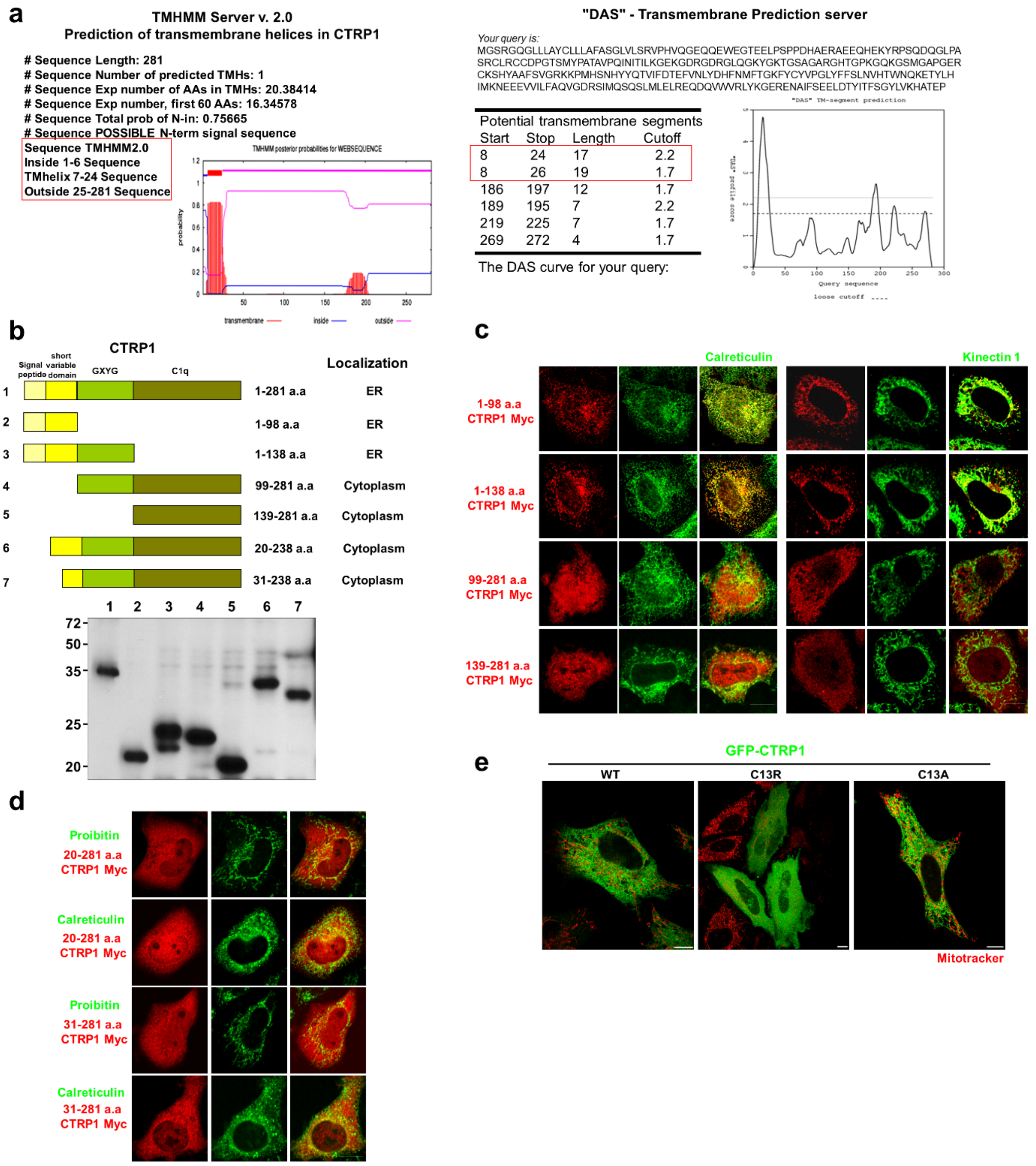

Supplementary Figure 3 CTRP1 has a transmembrane domain in its N terminus and is an ER membrane protein.

(a) Analysis of the CTRP1 transmembrane domain by the DAS and TMHMM programs.

(b) Schematic representation of a Flag-tagged CTRP1 mutant construct transfected into HeLa cells, and immunoblotting of these mutants with anti-Flag.

(c and d) HeLa cells transfected with Myc-His-tagged CTRP1 mutant constructs were immunostained with antibodies against the indicated proteins.

(e) MitoTracker staining of HeLa cells transfected with GFP-CTRP1 (WT), GFP-CTRP1 C13R or GFP-CTRP1 C13A. Scale bars, 10 μm.

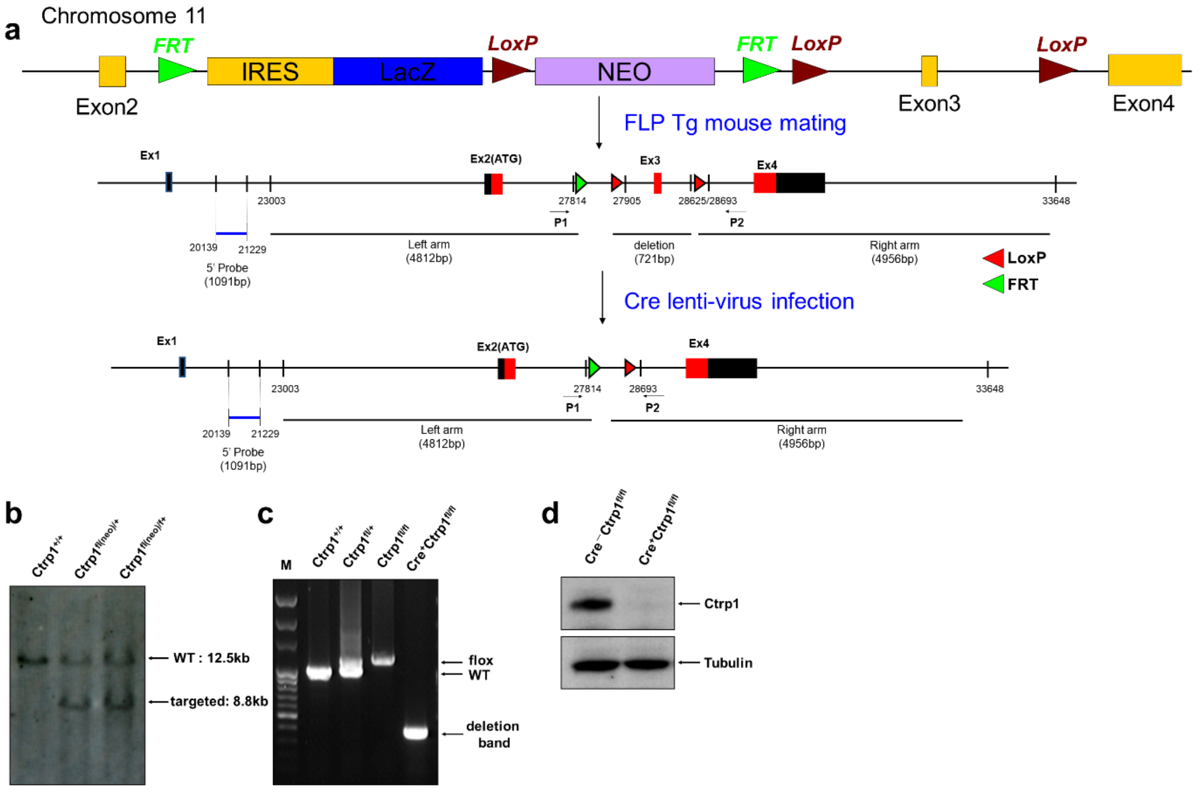

Supplementary Figure 4 Generation of a conditional *Ctrp1* knockout mouse.

(a) Schematic representations of the targeted *Ctrp1*$^{\text{fl}(neo)/+}$ cassette after its insertion into chromosome 11 by homologous recombination, the conditional *Ctrp1*$^{\text{fl/fl}}$ cassette after Flp-mediated recombination and the conditional *Cre*$^{+}$*Ctrp1*$^{\text{fl/fl}}$ cassette after Cre-mediated recombination. The selection marker (NeoR), expression marker (LacZ), coding exons (red bars), *FRT* and *loxP* sites (green and red triangles, respectively), external probes (blue boxes) and locations of the PCR primers (black arrows, P1, P2) are shown.

(b) Southern blot analysis of the targeted *Ctrp1*$^{\text{fl}(neo)/+}$.

(c) PCR analysis of DNA isolated from MEFs. DNA fragments amplified from wild-type (*Ctrp1*$^{+/+}$), floxed (*Ctrp1*$^{fl/fl}$) and knockout (*Cre*$^{+}$*Ctrp1*$^{fl/fl}$) mice are shown.

(d) Anti-CTRP1 was used to immunoblot MEF cells infected with control and Cre-encoding lentiviruses.

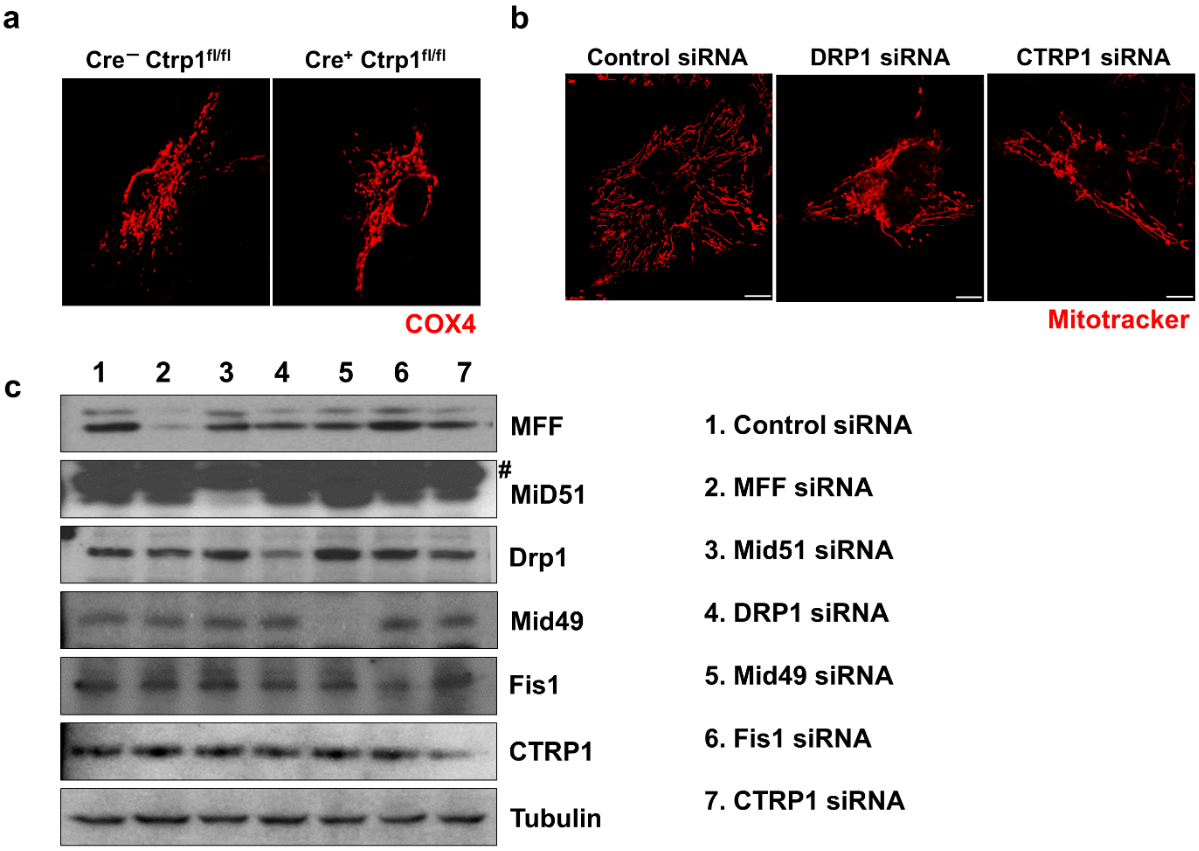

Supplementary Figure 5 CTRP1 is related to mitochondrial fission.

(a) $Cre^{-}Ctrp1^{fl/fl}$ and $Cre^{+}Ctrp1^{fl/fl}$ cells were stained with antibodies against COX4.

(b) Mitotracker staining of U2OS cells transfected with the indicated siRNAs.

(c) U2OS cells transfected with the indicated siRNAs were immunoblotted with antibodies against the indicated proteins. The crosshatch (#) represents nonspecific bands. Scale bars, 10 μm in a and b.

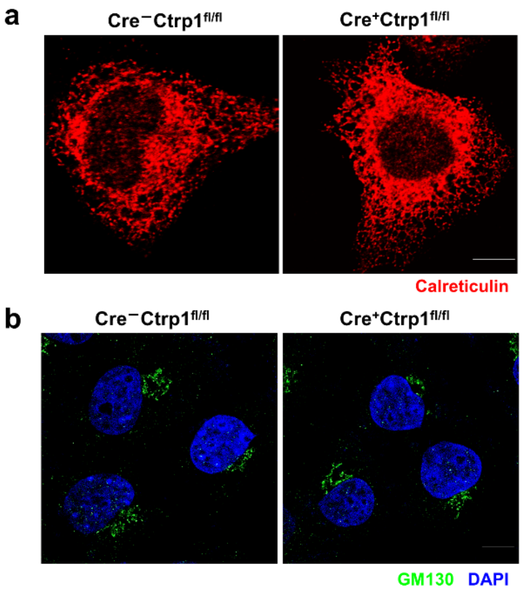

Supplementary Figure 6 Ctrp1 does not affect the morphology of the Golgi or ER.

(a) Cells immunostained with anti-GM130.

(b) Cells immunostained with anti-calreticulin. Scale bars, 10 µm.

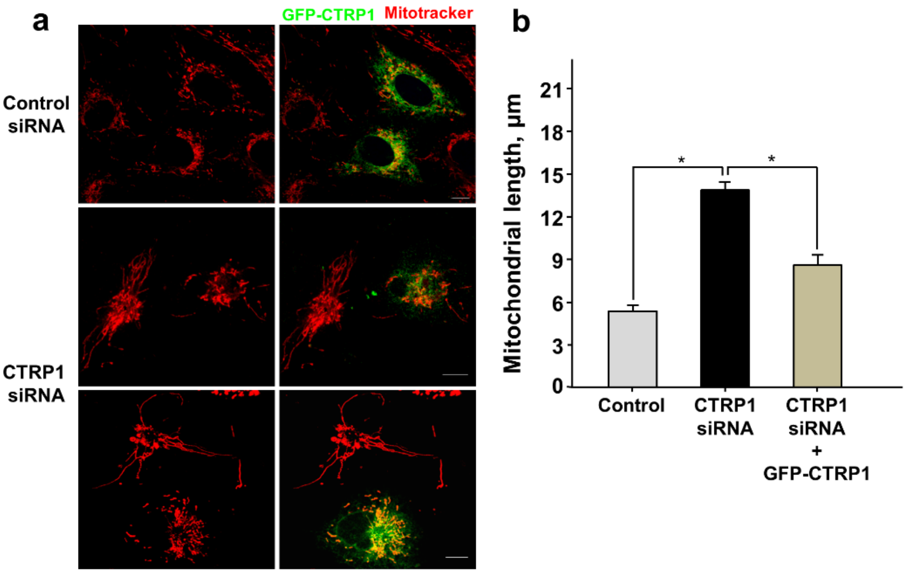

Supplementary Figure 7 Overexpression of CTRP1 partially rescues the defective mitochondrial morphology in CTRP1-knockdown U2OS cells.

(a) Mitotracker staining of U2OS cells transfected with the indicated siRNAs, GFP vectors or GFP–CTRP1.

(b) Quantification of mitochondrial lengths (n = 90 to 120 mitochondria). Data are presented as the means and s.e.m.*, P<0.001. Scale bars, 10 μm.

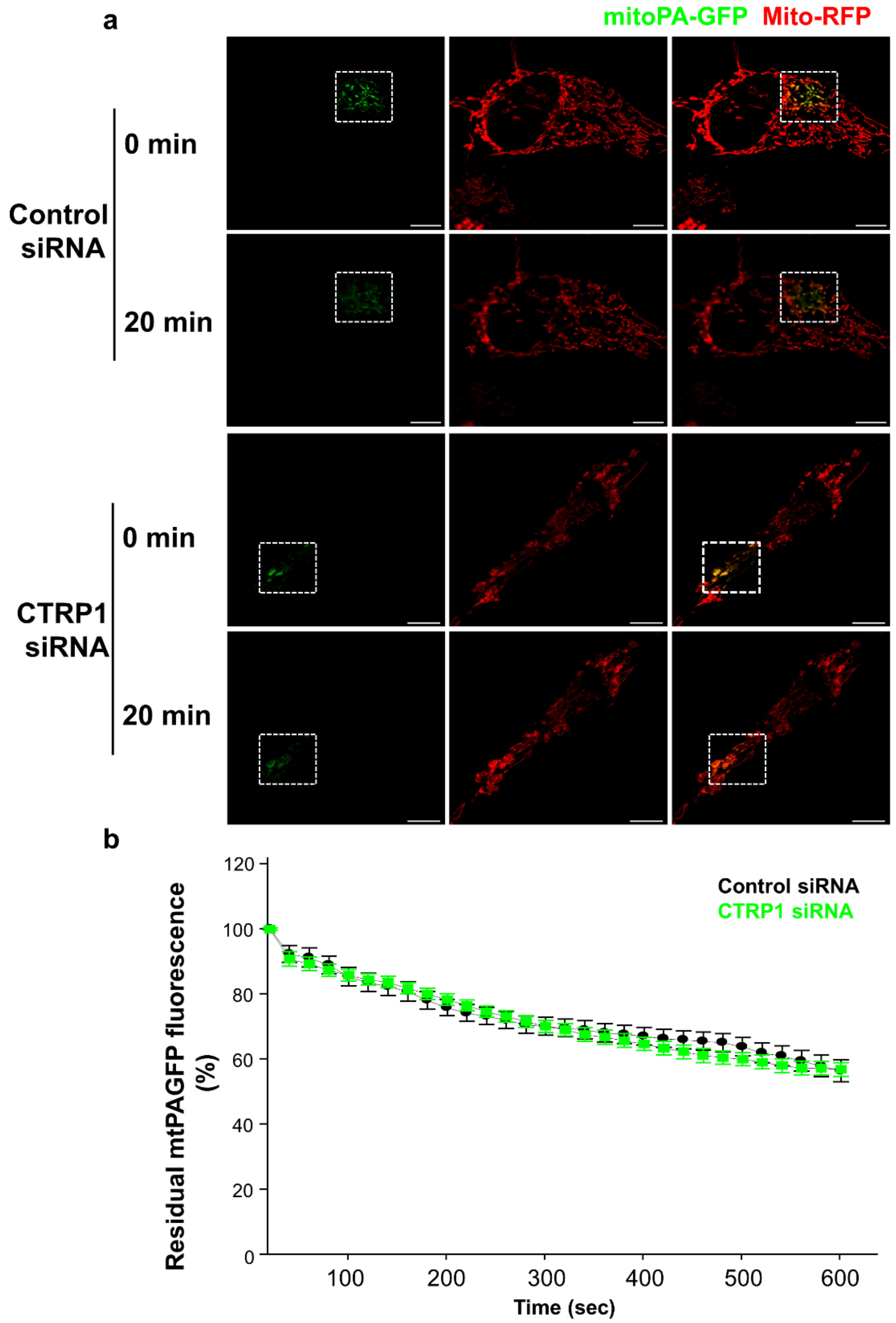

Supplementary Figure 8 Visualization of mitochondrial dynamics by mito-PAGFP in either control or CTRP1 siRNA treated Hela cells.

To activate the mito-PAGFP, regions of interest containing mitochondria were illuminated with a 405nm laser for 10 s and was then measured for GFP fluorescence every 20 s for 20 min.

(a) Representative images of Hela cells cotransfected with mito-RFP and mito-PAGFP after either control or CTRP1 siRNA treatment at the 0 or 20 min time point of 405nm illumination. Scale bar, 10 μm.

(b) Time course of GFP intensity in the initial region of interest following 405nm illumination. Data were presented as a relative percentage to the fluorescence in the 405nm illuminated region of interest. Mitochondria from regions of interest were measured in 10–15 cells per transfection. Error bars represent the S.E.M.

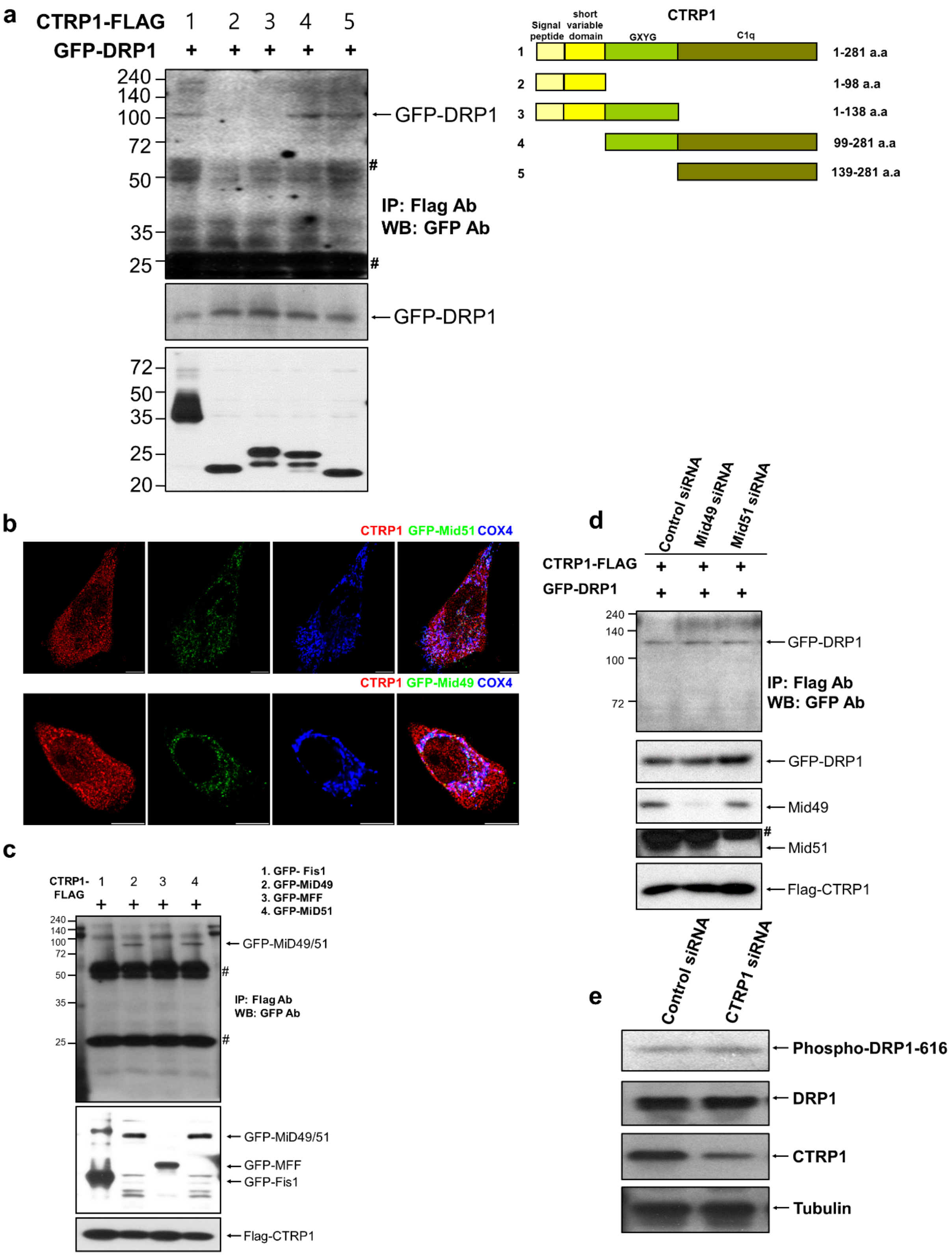

Supplementary Figure 9 C1q domain of CTRP1 interacts with Drp1 and CTRP1 interacts with the mitochondrial Drp1 adaptor, Mid49/51.

(a) Immunoprecipitation with anti-Flag beads and immunoblot with a GFP-specific antibody in 293T whole-cell lysates co-transfected with constructs expressing Flag–CTRP1 truncated mutants and GFP–DRP1. Asterisks indicate immunoglobulin G (IgG).

(b) Immunostaining of GFP-MiD49/51 transfected U2OS cells with antibodies against the indicated proteins. Scale bar, 10 μm.

(c) Immunoprecipitation with anti-Flag beads and immunoblot with anti-GFP antibody in 293T whole-cell lysates co-transfected with the indicated plasmids. The asterisks indicate immunoglobulin G (IgG).

(d) Immunoprecipitation with anti-Flag beads and then immunoblot with an anti-GFP antibody in 293T whole-cell lysates co-transfected with the Flag–CTRP1 and GFP–DRP1 after either control, Mid49 or Mid51 siRNA treatment. The asterisks indicate non-specific band

(e) Immunoblot with phosphor-DRP1 616, DRP1, CTRP1 and the cytosolic marker tubulin antibodies in U2OS cells transfected with the either control or CTRP1 siRNA.

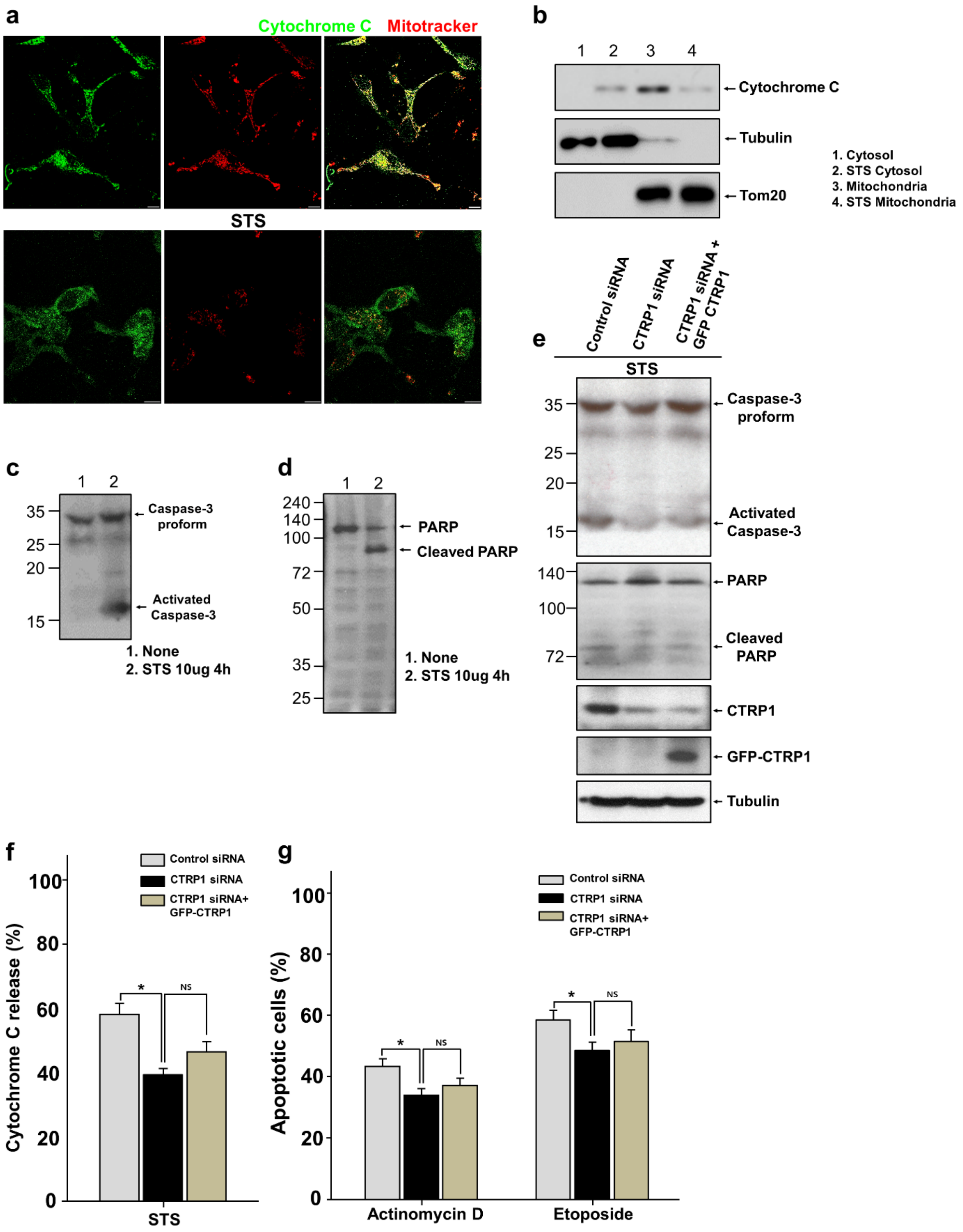

Supplementary Figure 10 Overexpression of CTRP1 generally rescues the apoptosis resistance in CTRP1-knockdown U2OS cells.

(a) Immunostaing of Mitotracker (red) and anti-cytochrome C (green) antibody in STS-treated U2OS cells.

(b) Immunoblot with cytochrome C, the cytosolic marker tubulin and the mitochondrial marker COX4 antibodies in STS-treated U2OS cells.

(c) Immunoblot with caspase-3 antibody in STS-treated U2OS cells.

(d) Immunoblot with PARP antibodies in STS-treated U2OS cells.

(e) Immunoblot with caspase-3, PARP and tubulin antibodies in U2OS cells transfected with GFP-CTRP1 and treated with STS after either control or CTRP1 siRNAs treatment.

(f) Quantification of cytochrome C release in U2OS cells transfected with GFP-CTRP1 and treated with STS after either control or CTRP1 siRNAs treatment(n = 300 cells).

(g**)** Quantification of fragmented nucleus in U2OS cells transfected with GFP-CTRP1 and treated with actinomycin D or etoposide after either control or CTRP1 siRNAs treatment (n = 300 cells). Data are presented as the means and s.e.m. Scale bars, 10 μm in a.

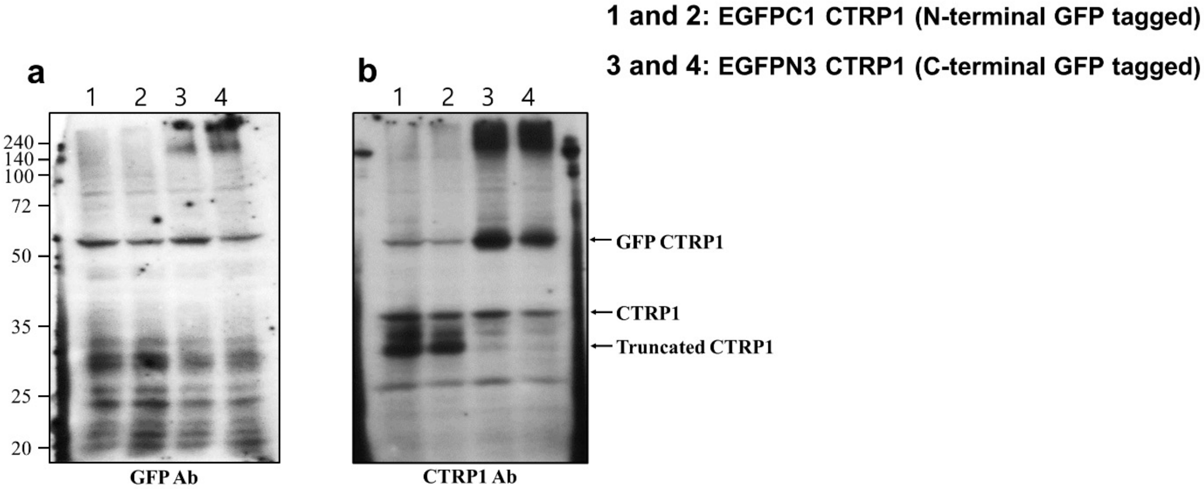

Supplementary Figure 11 CTRP1 may have membrane-bound and secreted forms.

(a and b) WCLs of 293T cells transfected with plasmids encoding EGFPC1 CTRP1 or EGFPN3 CTRP1 were immunoblotted (IB) with anti-GFP (a) or anti-CTRP1 (b).

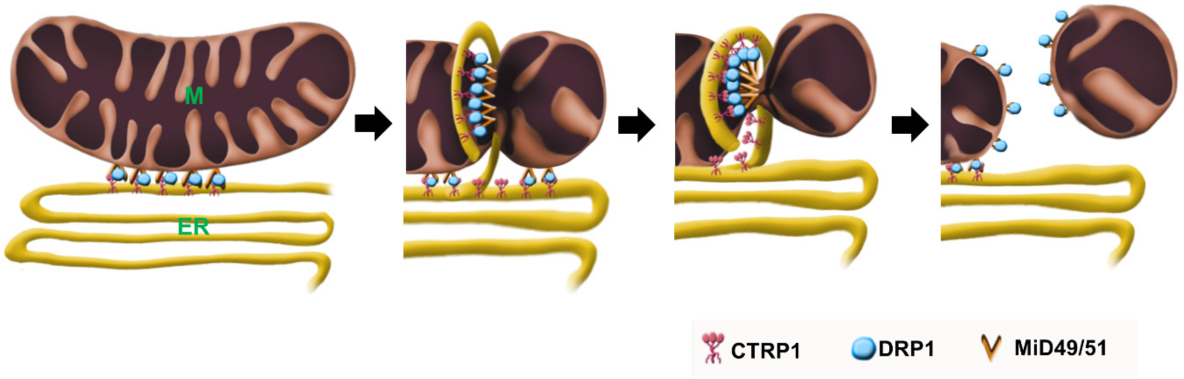

Supplementary Figure 12 A model showing the proposed function of CTRP1 during mitochondrial fission.

CTRP1 localizes to sites at which ER and mitochondria come into contact. Mitochondrial constriction begins through a DRP1-driven step at the mitochondrial fission site (MFS). ER-bound CTRP1 interacts with DRP1 at the MFS and surrounds the MFS. An interaction of ER-bound CTRP1 with DRP1 drives mitochondrial constriction until the mitochondrial fission event is completed.
